# Identification of Load Categories in Rotor System Based on Vibration Analysis

**DOI:** 10.3390/s17071676

**Published:** 2017-07-20

**Authors:** Kun Zhang, Zhaojian Yang

**Affiliations:** College of Mechanical Engineering, Taiyuan University of Technology, Taiyuan 030024, China; zhangkun0006@link.tyut.edu.cn

**Keywords:** rotor system, identification of load categories, back propagation neural network, ensemble empirical mode decomposition

## Abstract

Rotating machinery is often subjected to variable loads during operation. Thus, monitoring and identifying different load types is important. Here, five typical load types have been qualitatively studied for a rotor system. A novel load category identification method for rotor system based on vibration signals is proposed. This method is a combination of ensemble empirical mode decomposition (EEMD), energy feature extraction, and back propagation (BP) neural network. A dedicated load identification test bench for rotor system was developed. According to loads characteristics and test conditions, an experimental plan was formulated, and loading tests for five loads were conducted. Corresponding vibration signals of the rotor system were collected for each load condition via eddy current displacement sensor. Signals were reconstructed using EEMD, and then features were extracted followed by energy calculations. Finally, characteristics were input to the BP neural network, to identify different load types. Comparison and analysis of identifying data and test data revealed a general identification rate of 94.54%, achieving high identification accuracy and good robustness. This shows that the proposed method is feasible. Due to reliable and experimentally validated theoretical results, this method can be applied to load identification and fault diagnosis for rotor equipment used in engineering applications.

## 1. Introduction

Rotor systems are widely used in key machinery equipment of petrochemical, mining, and power and metallurgical industry, such as generator sets, centrifugal compressors, and similar applications. They are often subject to different types of loads during operation. For example, a rotating machine bears impact loads during rev. and stop, steady loads during smooth operation, and sinusoidal loads in case of misalignment and unbalanced failure. When reciprocating rolling plates, the roller system in a rolling mill is under transient load. Spindle rotor systems in winding hoists withstand linear load due to weight changes within the hoisting rope. Consequently, this also led scholars to research different types of load. For example, Hou et al. studied the nonlinear response and bifurcation analysis of a doffing type rotor model under sine load [1]. Bessam detected broken rotor bar faults in induction motor at low loads by using a neural network [2]. Husband studied the vibration response analysis of a typical turbofan engine under radial impact load [3], while Gui et al. studied resonance phenomena in shaft-bearing systems under impact, as well as sinusoidal, and rotational loads [4]. Thus, monitoring and identifying different load types is important. This has important theoretical and practical significance for analysis and diagnosis of rotor system running state, and guarantees safe and reliable operation.

However, direct measurement of parameters such as torque and stress would affect rotor dynamics; therefore, it is often not best suited for these applications. Instead, indirect load identification measurements are better suited and are a topic of active research [5]. In addition, these techniques provide accurate and reliable condition monitoring results for the systems. Load identification refers to techniques used to determine basic characteristics of dynamic loads in a system. This is based on known characteristics of a dynamic system and actual measurements of the resulting dynamic response. Load identification is an inverse problem of the second category for dynamic responses in a dynamic system [6]. The focal point of load identification in the study relates to non-stationary characteristics and system inversion. For this purpose, a system theory of linear and nonlinear, numerical computations, and computer simulations were conducted.

Several research groups are actively pursuing research in load identification due to the critical need to prevent catastrophic failure. Traditional methods of dynamic load identification mainly employ frequency and time domain based methods. A comprehensive review of frequency-domain identification methods is available [7]. Based on the research reported therein, it can be concluded that frequency domain methods are a relatively mature recognition method, based on the assumption of a linear system. For load identification, an inverse operation of feature matrices of the system is needed. Dynamic calibration is often simple. Due to this, these methods are in most cases easy to implement. For the frequency domain method, typically a signal with a certain length is requested. As a result, this method of load identification is not applied to nonlinear systems, but is generally applicable to steady state [8]. In addition, the frequency response function of the system always presents abnormal states near the resonance frequency, which leads to a significantly large identification error. Compared to traditional frequency-domain methods, time-domain identification methods are widely applicable for signal samples; therefore, many signals (including nonlinear and unstable) can improve the identification results by using this method. These are applied to deal with transient and impact load. Therefore, a time-domain method is more suitable and can be applied to nonlinear systems [9]. However, time-domain methods for load identification are sensitive to boundary and initial conditions of the structure, resulting in lower identification accuracy and increased robustness [10]. With the development of computer technology, some new algorithms have been introduced into the field of load identification, such as genetic algorithms, inverse pseudo excitation methods, and artificial neural networks. Among these algorithms, the operation of a genetic algorithm is simple, but comes with disadvantages such as prematurity, unsatisfactory global optimal value, and slow convergence speed [11]. The inverse pseudo excitation method (IPEM) is more suitable for steady loads. It also suffers from problems with noise interference and identification accuracy [12]. Neural networks used in artificial intelligence are promising for dynamic load identification [3]. Neural networks can be applied to various types of transient dynamic-loads and have high identification accuracy. Moreover, the identification model is stable, does not have any signs of divergence, or problems associated with cumulative error. A small amount of data is used to train the neural network, which allows much better identification compared to other methods. Additionally, this method has strong noise suppression, good robustness, and improved fault tolerance. Thus, neural networks provide an effective way to solve load identification in a non-linear and uncertain structural system [13]. Among ANN-style classifiers, there are several mature methods, such as support vector machine (SVM), radial basis functions neural network (RBFNN), and back propagation neural network (BPNN). These methods have good effects. For example, Yu et al. proposed the working condition diagnosis model based on SVM of submersible plunger pump [14]. Zhang et al. studied on the fault diagnosis technology of the water-projectile test posterior to the gun repair by RBFNN [15]. Li et al. effectively used neural networks to identify bearing faults on a motor system via measurement and interpretation of motor bearing vibration signatures [16].

Many vibration signals in rotor systems are relatively weak and have both non-linear and non-stationary characteristics. Therefore, prior to load identification, signal processing is required to highlight information characteristics. However, there are some problems in signal processing. For example, short-time Fourier transform (STFT) can only add the same “window function” for changing signals in different periods. Consequently, it is only applicable for analyses of slow-changing signals [17]. Wavelet analysis demands smoothness of the “window signal” and has shortcomings of signal energy leak and non-adaptivity [18]. Empirical mode decomposition (EMD) is an effective signal processing method for non-linear and non-stationary signals. Due to its good local characteristics and adaptability, this method has an effective application in many fields such as seismic analysis, and fault diagnosis [19,20]. However, modal mixings are in EMD method, especially when original signals contain interstitial or pulse components. The EEMD method can improve this problem and obtain better information characteristics. It offers better sample data for neural networks, and prepares for the next step of load identification. 

Modern load identification methods have rarely been applied to a rotor system in rotating machinery, and identification of rotor systems for various load types is uncommon. In this study, based on these typical load conditions in a rotor system, five typical types of loads have been refined for study, namely impact load, steady load, linear load, sinusoidal load, and transient load [1,2,3,4]. Specifically, an identification method for load categories is proposed here based on vibration signals of the rotor system. This method combines ensemble empirical mode decomposition, energy feature extraction, and BP neural network. Meanwhile, the BP Network is of reasonable construction here, achieving high identification accuracy and good robustness.

## 2. Methodologies of Vibration Signal Based on Load Category

**Figure 1:**
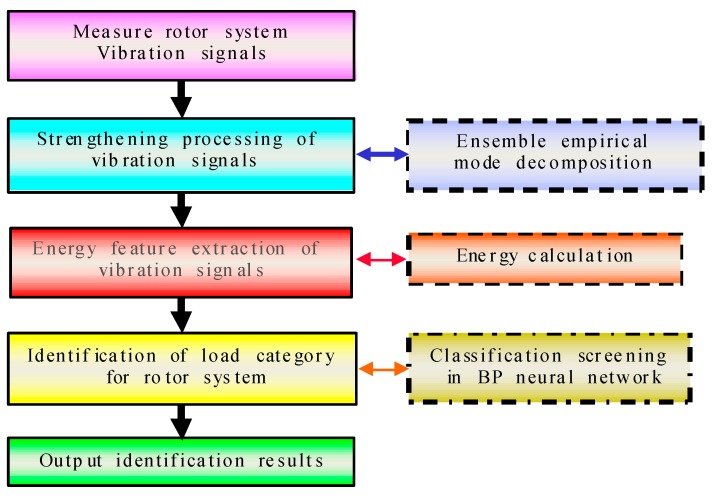
Flowchart of identification method of load categories for rotor system.

A flow chart showing the steps involved in the method for identification of rotor system load category is shown in Figure 1. Five main steps were utilized for the identification method, which are shown in the left half of the figure. First, load vibration signals were imported. This was followed by a comprehensive pre-processing of the vibration signal, energy feature extraction, and identification of load category for rotor system, thus finally obtaining identification results. On the right side of the diagram, the first textbox at the top represents process decomposition of pre-treatment for the vibration signal. This is the ensemble empirical mode decomposition. Both textboxes below represent the identification of load category for rotor system, using energy calculation and BP neural network method for classification screening. The two steps are achieved based on signal pre-processing.

### 2.1. Vibration Signatures Enhancement Based on EEMD

**Figure 2:**
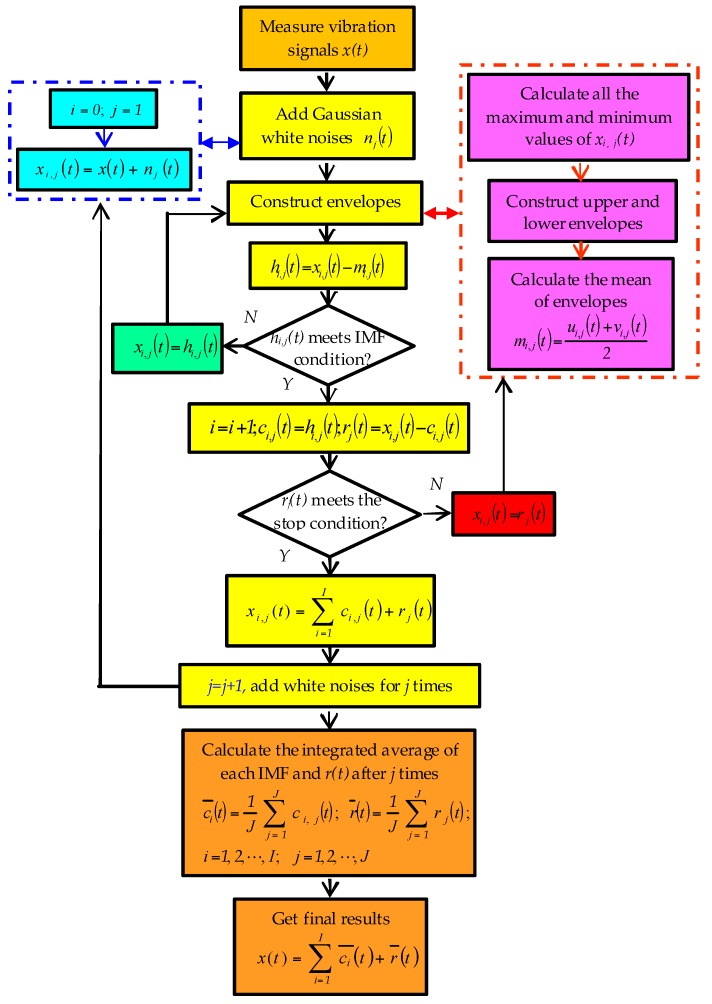
Flowchart of EEMD.

EEMD is an improved method, which was proposed to solve problems associated with pattern aliasing problems of the EMD method. It is a noise-assisted data analysis (NADA) method [21]. The decomposition principle is based on the fact that signal areas in different scales are automatically mapped to appropriate scales associated with white background noise with uniform distribution. After averaging several times, the joined Gaussian white noises cancel each other out, thus restoring the original signal. The signal is the only persistent part and multiple tests allow noise to be eliminated. Figure 2 shows a flowchart of the EEMD method, illustrating the steps followed.

In Figure 2, the steps of EEMD method are as follow:

1. Some Gaussian white noise sequences are added to the original signal respectively. The *j*-th white noise adding is taken as an example. A white noise *n_j_*(*t*) is added to the original signal *x*(*t*), which is expressed as:
  (1) $$x_{i,j}\left(t\right)=x\left(t\right)+n_{j}\left(t\right)\;i=0,1,\cdots ,I;\;j=1,2,\cdots ,J$$
  In the equation, *n_j_*(*t*) means adding the *j*-th white noise. *j* means the sequence of adding white noise. *J* denotes the total number of additions of white noise. *x_i_*_,*j*_(*t*) represents the *i*-th order signal decomposed after the *j*-th adding noise. *i* expresses the sequence of intrinsic mode function (IMF) components obtained via EMD after addition of one noise. *I* is the total number of IMFs.
2. Then, *x_i_*_,*j*_(*t*) is decomposed. During this process, maximum and minimum points of the signal are calculated. Next, upper and lower envelopes of the signal are constructed, and the average *m_i_*_,*j*_(*t*) is calculated.
  (2) $$m_{i,j}\left(t\right)=\frac{u_{i,j}\left(t\right)+v_{i,j}\left(t\right)}{2}$$
  In the equation, *u_i_*_,*j*_(*t*) and *v_i_*_,*j*_(*t*) mean upper and lower envelopes, respectively.
3. The average signal *m_i_*_,*j*_(*t*) is subtracted from the original data to yield *h_i_*_,*j*_(*t*), a new sequence without low frequency components removed.
  (3) $$h_{i,j}\left(t\right)=x_{i,j}\left(t\right)-m_{i.j}\left(t\right)$$
  Judging whether *h_i_*_,_*_j_*(*t*) satisfies two IMF-conditions uses the whole data sequence, and sets the number of extreme value points equal to the number of passing zero points. The maximum difference of two numbers cannot be above one point. At any point, the mean of the envelopes is zero, which is defined by the maximum and minimum local values of signals. If *h_i_*_,_*_j_*(*t*) does not meet the IMF condition, the calculation process needs to be repeated. If the criterion is met, this is the first IMF component *c_i_*_,*j*_(*t*). This is the highest frequency component of the signal sequence. *c_i_*_,*j*_(*t*) subtracted from *x_i_*_,*j*_(*t*) provides the new data sequence *r_j_*(*t*).
  (4) $$i=i+1;c_{i,j}\left(t\right)=h_{i,j}\left(t\right);r_{j}\left(t\right)=x_{i,j}\left(t\right)-c_{i,j}\left(t\right)$$
  Next, *r_j_*(*t*) is checked against the stop condition. Namely, when *r_j_*(*t*) becomes a monotone function, the screening ends. If the stop criterion is not met, *r_j_*(*t*) needs to be calculated again using the above-mentioned decomposition. This process is repeated until the last data sequence cannot be decomposed any further.
  (5) $$x_{i,j}(t)=\sum_{i=1}^{I}c_{i,j}\left(t\right)+r_{j}\left(t\right)$$
  In the formula, *c_i_*_,*j*_(*t*) is the *i*-th IMF component after EMD, when adding the *j*-th white noise, while *r_j_*(*t*) is the residual component after EMD, when adding the *j*-th white noise.
4. Step 1. is repeated *J-1* times. For each iteration, a new sequence of Gaussian white noise *n_j_*(*t*) is added. During EEMD, the total times of Gaussian white noise obey the statistical regularity of Equation (6).
  (6) $$\varepsilon_{n}=\frac{\varepsilon }{\sqrt{J}}$$
  In the equation, ε is the amplitude of Gaussian white noise and ε*_n_* denotes the error between the initial signal and the sum of IMFs. According to the paper [22], when *J* is between 100 and 300, the residual white noise error has been at a low level.
5. After adding white noise for *J* times, for a given IMF, intrinsic mode components are calculated using integrated average processing.
  (7) $$\bar{c_{i}}\left(t\right)=\frac{1}{J}\;\sum_{j=1}^{J}\;c_{i,j}\left(t\right);\;i=1,2,\cdots ,I;\;j=1,2,\cdots ,J$$
  Meanwhile, after *J* times decomposition, residual components *r_j_*(*t*) are calculated using an integrated average processing as well.
  (8) $$\bar{r}\left(t\right)=\frac{1}{J}\sum_{j=1}^{J}\;r_{j}\left(t\right)$$
  Thus, final results are obtained.
  (9) $$x(t)=\sum_{i=1}^{I}\bar{c_{i}}\left(t\right)+\bar{r}\left(t\right)$$
  In the formula, $\bar{c_{i}}\left(t\right)$ represents each IMF component, which is finally obtained after EEMD. $\bar{r}\left(t\right)$ is the final residual component.

### 2.2. Energy Feature Extraction of IMF Vibration Signal

A signal with added white noise is processed each time via EEMD to obtain the corresponding IMF. Subsequent to EEMD, each IMF represents a stationary signal with a set of characteristic scales. Change in energy at each frequency band represents characteristics of different loads. Energy and residual component of each component can be obtained via signal decomposition. Residual component does not contain any useful information. Any useful information in the residual component is considered negligible. Due to orthogonality of the decomposition, the sum of IMF energies is identically equal to the total energy of the signal. The steps of energy extraction are as follows:

1. From the EEMD of the signal, select I IMF components containing information of the load.
2. Solve for energy value *E_i_* of each *IMF_i_*. Here *IMF_i_* is equal to $\bar{c_{i}}\left(t\right)$ in Equation (7).
  (10) $$E_{i}={\int_{-\infty }^{+\infty }\left|IMF_{i}\right|}^{2}dt\;(i=1,2,\cdots ,\;I)$$
3. Construct a feature vector **E** formed with energy.
  (11) $$E=\left[E_{1},E_{2},\cdots ,E_{I}\right]$$
  The obtained energy ratio is defined as follows:
  (12) $$T_{i}=E_{i}/\sum_{i=1}^{I}E_{i}$$
  When different loads are applied to the rotor system, displacement signal energy changes in each frequency band. Energy in each frequency band contains information related to loading. Therefore, IMF energy components from the signals are extracted as feature vectors of load type.

### 2.3. Classification Method

#### 2.3.1. Choice of the Classifier

There are several good classifiers, such as support vector machine (SVM), radial basis functions neural network (RBFNN) and back propagation neural network (BPNN). SVM is to establish a classification hyperplane as decision surface. RBFNN and BPNN belong to the feedforward neural network in the network structure. When choosing the classifier in the study, these were calculated and compared. The same sample data were used in these methods, which from the measured signals of load identification experiment in rotor system, shown in “3. Load category Identification Experiments”. The load classification effects in three methods are as shown in Table 1.

In Table 1, RBFNN is the fastest in the operation time. To achieve early stopping, the training functions with fast convergence were not used in BPNN, which caused a time consuming. The running time in SVM is in between. SVM and BPNN are with high accuracy, and the latter is slightly higher. Overall, these three methods can all satisfy the requirements of load classifications in rotor system. Here, BPNN is chosen in this paper.

#### 2.3.2. Classification with BP Neural Network

The core idea of a BP neural network algorithm is to feed output from error back-propagation back into the input layer though a hidden layer. It is composed of two processes: forward-propagating (forward calculation) of data flow, and back-propagation of error signal. By alternating between both processes, a gradient descent strategy of the error function is executed in the weight vector space. Dynamic iterations are conducted to screen a group of weight vectors. Due to this, the network error function reaches a minimum. At this time, the information processes are assumed to be completed [23]. The training process of the BP neural network includes the following steps:

1. Network initialization. According to input and output sequence (*x*, *y*) of the system, the network needs to determine the number of input layer nodes n, the number of hidden layer nodes l, and the number of output layer nodes m. Then, connection weights *ω_ij_* and *ω_jk_* are initialized for neurons of input, hidden, and output layers. Hidden layer threshold a, and output layer threshold b are initialized. Learning rate and excitation function of neurons are specified. Number of hidden-layer nodes for the BP neural network can be designed based on empirical Equation (13). *p* is an adjustable integer constant between 1–10.
  (13) $$l=\sqrt{n+m}+p$$
2. Output calculation of the hidden layer. Based on input variable *x*, connection weights *ω_ij_* between input layer and hidden layer, and hidden layer threshold *a*, the hidden layer yields output *H*.
  (14) $$H_{j}=f\left(\sum_{i=1}^{n}\omega_{ij}x_{i}^{}-a_{j}\right)\;j=1,2,\cdots ,l$$
  In this formula, *f* is the excitation function of the hidden layer. This function has a variety of forms. One of these forms has been chosen below.
  (15) $$f\left(x\right)=\frac{1}{1+e^{-x}}$$
3. Output calculation for output layer. According to hidden layer output *H*, connection weight *ω_jk_* and threshold value *b*, BP neural network calculates the predicting output *O*.
  (16) $$O_{k}=\sum_{j=1}^{l}H_{j}\omega_{jk}-b_{k}\;k=1,2,\cdots ,m$$
4. Based on the predicting output *O* and desired output *y*, prediction accuracy for the network is evaluated.
5. Connection weights *ω_ij_* and *ω_jk_* in the network are updated.
  (17) $$\omega_{ij}=\omega_{ij}+\eta H_{j}\left(1-H_{j}\right)x\left(i\right)\sum_{k=1}^{m}\omega_{jk}\left(y_{k}-O_{k}\right)\;i=1,2,\cdots ,n;j=1,2,\cdots ,l$$
  (18) $$\omega_{jk}=\omega_{jk}+\eta H_{j}\left(y_{k}-O_{k}\right)\;j=1,2,\cdots ,l;k=1,2,\cdots ,m$$
  η indicates the learning rate.
6. Next, node thresholds *a* and *b* in the network are updated.
  (19) $$a_{j}=a_{j}+\eta H_{j}\left(1-H_{j}\right)\sum_{k=1}^{m}\omega_{jk}\left(y_{k}-O_{k}\right)\;j=1,2,\cdots ,l$$
  (20) $$b_{k}=b_{k}+\left(y_{k}-O_{k}\right)\;k=1,2,\cdots ,m$$
7. Check if stop condition for the iterations is reached. If not, go to step 2.

**Figure 3:**
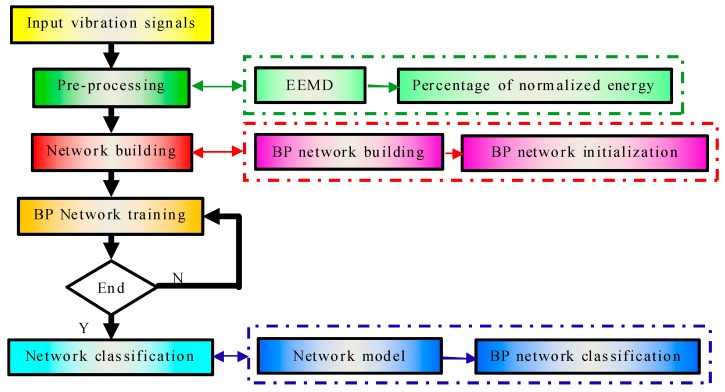
Flowchart combining EEMD and BP neural network.

Design of a suitable BP neural network, followed by training the network, allows for realization of qualitative load identification of the rotor system. Data from EEMD feature extraction is input into the neural network. The data contains abundant load information. The BP neural network acts as classifier, and then the network can be set up and trained. The established BP neural network has the ability to learn. Test data are classified through the network, and then different types of loads can be identified. The algorithm process is combined with EEMD and BP neural network, as shown in Figure 3.

## 3. Load Category Identification Experiments

### 3.1. Experimental Program

According to the research objective, experiments were designed for load identification. A special test bench of was designed to allow load category identification of the rotor system. Every time the rotor system speed is increased up to a rated speed of 1480 r/min, five types of torque are applied to the rotor system in turn, via programmed control system of torques. For each test vibration, signals were collected for only one excitation torque applied to the rotor system. According to each measured vibration signal of the rotor system, triggered by the corresponding type of load, load category identifications of the rotor system can be conducted.

There are five types of loads designed and applied in the experiments: impact load, steady load, linear load, sinusoidal load, and transient load. Impact load refers to a load changing rapidly as a function of time. In this case, time required for the load value to increase from zero to a maximum is less than half of the natural vibration period applied to the impacted body. Steady load is held constant and always maintains a stable state, i.e. load value does not change over time. Linear load refers to a linear change in load as a function of time. For a sinusoidal load, load changes over time following the sine curve. Transient load refers to change in load in a short time period relative to total time. This change in load often lasts for several seconds. In the experiment, five types of load values are as shown in Table 2. In the load expression table, *M* is the load torque, with unit of Nm, and *t* is the time in s.

Loads of different parameters are selected randomly to be identified here, namely, impact loads (40 Nm, 60 Nm, 80 Nm) for 0.5 s, steady loads (40 Nm, 60 Nm, 80 Nm), linear loads (0.1 t + 40 Nm, 0.2 t + 40 Nm, t + 40 Nm), sinusoidal loads (sin10πt + 40 Nm, 20sin4πt + 60 Nm, 10 sin4πt + 40 Nm) and transient loads (40 Nm, 60 Nm, 80 Nm) for 3 s.

### 3.2. Test Platform Build

**Figure 4:**
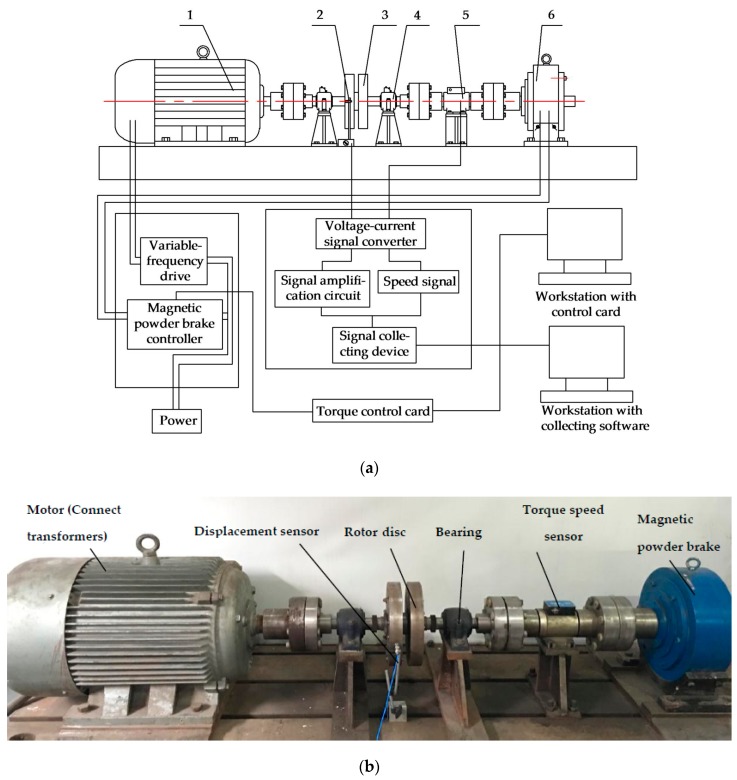
Load-identification test bench of rotor system. (**a**) Design schematic: 1—motor; 2—eddy current displacement sensor; 3—rotary disc; 4—bearing; 5—torque speed sensor; 6—magnetic powder brake; (**b**) Main part of load-identification test bench.

In general, this paper focuses on the rotor system. To simulate real working conditions, and combined with experimental requirements, a rotor system test bench for dynamic loading was designed and built. This system is based on vibration signals, rather than on ordinary small teaching rotor beds. A design schematic of the test bench for load identification and photograph of the main working part of the proposed test bench are shown in Figure 4. For certain working conditions, experimental load identification was conducted on the bench. Five different types of torque were designed for the experiments. In this way, both normal and loading states can be simulated for a rotor system. Vibration signals on the test bench were measured using installed eddy current displacement sensors in the rotor system.

The designed test bed is mainly composed of four parts: a mechanical unit in the rotor system, a variable frequency (speed control) unit, a torque actuator unit, and a signal test unit. In the mechanical unit of the rotor system, a three-phase asynchronous motor powers the mechanical parts of rotor system. According to requirements of the test, motor power is designed as 55 kW, rated speed is 1480 r/min, while deep groove and ball bearings 6308 serve as supports. The variable frequency (speed control) unit is composed of a frequency converter and its auxiliary components. Two sets of control circuits are designed, for automatic and manual controls. This can adjust the converter output frequency manually and continuously, start in established procedures, and run at a specified frequency. The torque control unit can furnish the rotor system with different types and sizes of dynamic torque loads, through both control card and brake. While carrying out these experiments, it is important to note that the magnetic powder brake within the torque process control system, needs to be ensured that the continuous use of time does not extend beyond 5 min. The signal test unit mainly consists of eddy current displacement sensors, signal acquisition system, and software. It is used to monitor displacement vibration of rotary disc in rotor system. In the experiment, vibration signals were tested and analyzed under different dynamic loads. Related calculations have been conducted with MATLAB (The Mathworks, Inc. Natick, MA, USA).

## 4. Analysis of Experimental Results

### 4.1. Comprehensive Pre-Processing of Load Vibration Signal

After five different successive loading experiments of the rotor system, displacement vibration signals along the radial horizontal direction for rotary disc are collected. For example, when a periodically sinusoidal torque M = (10 sin10πt + 40) Nm was applied for 10 s, the corresponding displacement vibration signals in radial horizontal direction are acquired. Similarly, vibration signals of the rotary disc were tested with other load types.

If the original vibration signals are fed into the network for classification and recognition, characteristics of the signals were not prominent and would need long processing times to identify characteristics [24]. After EEMD, original signals are decomposed into different frequencies. The mixed component of frequency spectrum can be differentiated from high to low frequencies. Here, the measured vibration signals along radial horizontal direction of rotary disc are processed via EEMD. This is subsequently followed by extraction of energy features.

**Figure 5:**
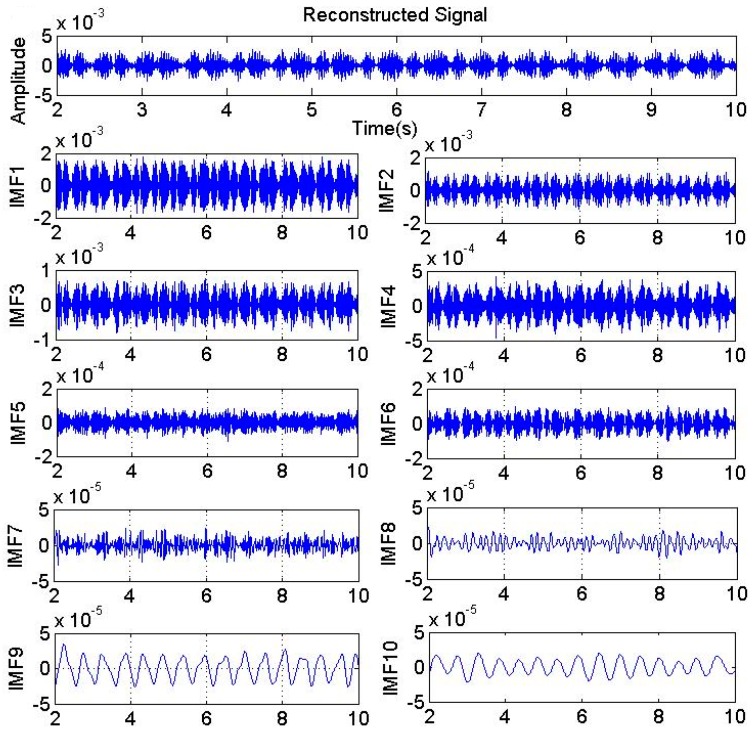
Ensemble empirical mode decomposition.

Sinusoidal load M = (10 sin10πt + 40) Nm from the previous section is taken as an example here. In EEMD, the selected Nstd was 0.02, while Ne was 100. Next, IMF components with an order of 14 and a residual component were obtained. Figure 5 shows the effect of EEMD for vibration signals, listing IMFs in top 10 orders. It can be seen from the decomposition process, that after EEMD, IMF1–IMF10 contains components of the sine signal in different time scales from small to large. IMFs contain components of the signal at different frequencies, from high to low frequencies. The order is inversely proportional to the frequency of the components. The EEMD method can be seen as a set of bandpass filters with adaptive characteristics. This is because after EEMD decomposition, IMF components have different frequency components and bandwidths. Furthermore, frequency components and bandwidths vary with the decomposed signal and IMF component, which preferentially decomposed high frequency components.

At the same time, processing methods of vibration signals corresponding to other load types are similar to the sine load. As a result, processing does not have to be repeated.

### 4.2. Energy Feature Extraction of Load Vibration Signal

After EEMD, the energy of each signal component is extracted as a feature vector of the load type. For example, five types of loads are randomly selected, namely, impact load (40 Nm) for 0.5 s, steady load (40 Nm), linear load (t + 40 Nm), sinusoidal load (10 sin10πt + 40 Nm), and transient load (40 Nm) for 3 s.

**Figure 6:**
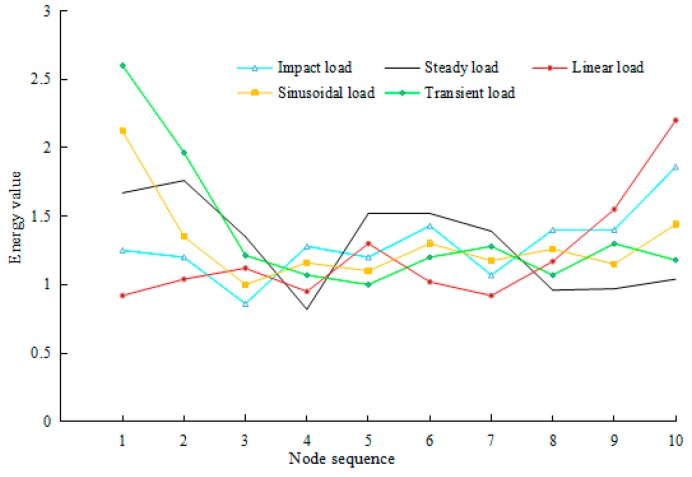
Energy distribution of nodes.

For these five load types in the rotor system, IMFs of the corresponding vibration signals are implemented for an energy feature extraction. After energy calculation and normalization, it is divided into 10 “energy nodes” based on energy calculation and energy changes of vibration signals for each corresponding load type are obtained. These energy changes are embodied in the node energy distribution, as shown in Figure 6. The abscissa shows the represent node sequence and ordinate expresses the energy value. Each load curve represents energy change corresponding to a load type, according to the order of nodes.

It can be seen from the energy graph shown in Figure 6 that different load types have different energy values. Additionally, fluctuations in energy values for different loads are not identical. For IMF components within the same order, vibration signals contain different frequency components and have different energy values, under different load cases. Therefore, energy feature extractions of IMF components can be used for load classification. Through energy extraction, a large number of data are integrated using few characteristics data for ease of analysis as well as to prepare for subsequent load identification.

### 4.3. Category Identification of Experimental Load

The BP neural network is an adaptive nonlinear dynamic network, composed of a large number of connected neurons. Here, the physical mechanism of information processing of the human brain is simulated. This allows the network to solve problems, ratiocinate, and learn. Input and output data are often included for most dynamic characteristics in an engineering structure. Thus, an artificial neural network can be applied for load identification. Thus, after training, an artificial neural network model can map nonlinear relationships between vibration responses of the structure, dynamic loads, and weights among neurons. This method compared to the traditional method does not need to establish theoretical expressions between vibration responses and dynamic loads. This method has higher modeling accuracy, error control, and realistic operability.

#### 4.3.1. BP Neural Network Training

Here, the used neural network is a general function approximator with three layers, formed by three layers of BP neural network, including the input layer, the output layer, and the hidden layer [25]. This setup used here is the most common structure. The number of feature vectors is 10 for each signal, selected after EEMD; therefore, the input dimension of characteristic vectors is 10. To identify five load types, the node-number of the input layer is 10, and the dimension of the output layer is 5 for the final design of BP neural network. Neural network sometimes may overfit during the training. That is to say, the identification error for training set is very small, while for testing set is too big. It can be mainly considered as the following points, so as to guarantee the robustness of this method [13].

##### Number of Hidden Layer Nodes (NHLN) Setting

If NHLN is too small, the network can neither study well nor identify unknown samples. Its fault tolerance is poor, and the training error is big. If the nodes are too many, it will cause a longer training time, and even an overfitting. Thus, NHLN can affect the network precision and the system robustness. NHLN needs to be chosen. Table 3 shows the influence of the load identification results by NHLN in BP network. According to Equation (13), the initial numbers of nodes in the hidden layer range from 5 to 14. As shown in Table 3, network error and training times are obtained after training BP neural network in different numbers of nodes. When the number of hidden layer nodes is 12, a smaller error is achieved with fewer training times. Therefore 12 was chosen for the number of nodes in the hidden layer.

##### Node Transfer Function (NTF) Selection

NTF selection can influence testing precision in network. Here, NTF in hidden layer is logsig, which can make an easy convergence for network during the training process. While NTF in output layer is purelin. There is no limit on the size of output values. Meanwhile, in other same conditions, the combination of the two functions tends to have high prediction accuracy.

##### Network Training Function Selection

Different training function can affect the network performance. In order to achieve early stopping and ensure the network robustness, trainscg is selected to train network. This algorithm can avoid consuming huge time for search process. Especially when the expected network error is small, the algorithm is still reliable.

Types of torque loads are outputs. For network coding of load types, when the expected output is 1, this indicates that the system is in the current load condition. For the five load types, classification codes correspond to mathematical codes of the expected output, as shown in Table 4. The selected learning rate is 0.01, inertia coefficient is 0.2, training time is 5000, and training error is 10^−3^. In the training, weights and threshold values are adjusted according to the error of network training.

The corresponding vibration signals were measured in the experiment (Table 2). Training sample data were obtained after using EEMD and energy feature extract for signals, while the rest of the data are texting samples. Table 5 lists part of the training sample data corresponding to five types of loads.

#### 4.3.2. Load Type Identification in BP Neural Network

After training, weights and threshold values are preserved. At this time, the BP network has an ability to store and predict values. Table 6 lists part of the testing sample data. Data can be input into the trained network model, and then the network will commence screening for the input data in accordance with the model. Finally, automatic classification and identification of loads can be completed.

**Figure 7:**
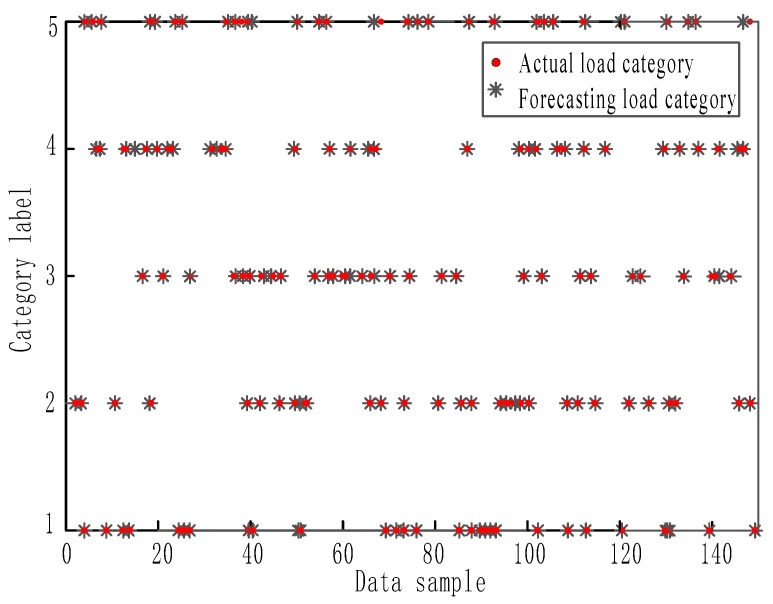
Prediction effect of BP network.

After processing with the BP neural network classifier, predictions are shown in Figure 7. It shows the comparison of forecasting and actual situation of 150 testing sample data. In Figure 7, the abscissa is the testing data sample and the ordinate shows identifying codes (1–5) for the five load types. It can be observed that for the various load conditions, predicted data are identical to actual data.

After verification of these data, identified loads are compared with original test loads. For the five load types (impact, steady, linear, sinusoidal, and transient), the correct recognition rate is 90.91%, 95.45%, 90.91%, 95.45%, and 100% respectively; therefore, the total recognition rate is 94.54%. Identification errors were found to be within the permitted range. After the reasonable settings of the parameters, the identifications were conducted only for the non-training samples. Therefore, final testing samples obtained high identification accuracy. It shows that this method is feasible and with a robustness.

## 5. Conclusions

For five different loads types, a method using EEMD is proposed to analyze vibration signals for rotor systems. Using EEMD as a signal pre-processor, original signals can be decomposed into different frequencies, and mixed components of the frequency spectrum can be differentiated from high to low frequencies. Therefore, EEMD is used to reconstruct and decompose original signals to further extract subsequent characteristic signals.

After extracting the feature signal energy, a large data set can be reduced in size. The trend of characteristic energy for each load type is different to the other. In this way, the feature of each load can be distinguished, thus providing accurate data sources for qualitative load identification.

A method of load identification in rotor systems, based on EEMD, is proposed and classification screening was conducted using the BP neural network. Feature energy for full load information is input into the trained model of BP neural network. With the ability for storage and prediction in network, feature information is screened to achieve qualitative load identification. The total recognition rate is 94.54%, with good identification accuracy. This demonstrates feasibility of the method. It also provides a theoretical basis and experimental verification for dynamic design, load identification, and operation monitoring in a rotor system.

## Figures and Tables

**Table 1:** Load classification effects in three methods.

Classifier	Time (s)	Accuracy (%)
SVM	2.215	92.071
RBFNN	0.633	83.182
BPNN	3.218	94.550

**Table 2:** Loads in rotor-system experiment.

**Load Type**	**Expression (Nm)**			
impact (0.5 s)	M = 40	M = 50	M = 55	M = 60
steady	M = 40	M = 50	M = 55	M = 60
linear	M = 0.1 t + 40	M = 0.1 t + 50	M = 0.2 t + 40	M = 0.2 t + 50
sinusoidal	M = sin4πt + 40	M = sin4πt + 60	M = sin10πt + 40	M = 10 sin4πt + 40
transient (3 s)	M = 40	M = 50	M = 55	M = 60
**Load Type**	**Expression (Nm)**			
impact (0.5 s)	M = 65	M = 70	M = 75	M = 80
steady	M = 65	M = 70	M = 75	M = 80
linear	M = 0.5 t + 50	M = t + 30	M = t + 40	M = t + 50
sinusoidal	M = 10 sin4πt + 60	M = 10 sin10πt + 40	M = 20 sin4πt + 60	M = 20 sin10πt + 60
transient (3 s)	M = 65	M = 70	M = 75	M = 80

**Table 3:** Training error and times of BP neural network (in different numbers of nodes for the hidden layer).

**Number of Nodes**	**5**	**6**	**7**	**8**	**9**
Error (10^−9^)	9.996	9.961	7.705	9.962	9.571
training times	990	972	508	290	292
**Number of Nodes**	**10**	**11**	**12**	**13**	**14**
Error (10^−9^)	1.297 × 10^−^^2^	2.511 × 10^−1^	4.463 × 10^−^^3^	2.526	8.881 × 10^−1^
training times	156	72	77	90	86

**Table 4:** Codes of five types of loads.

Load Type	Impact	Steady	Linear	Sinusoidal	Transient
sorting code	1	2	3	4	5
expected output	[1 0 0 0 0]	[0 1 0 0 0]	[0 0 1 0 0]	[0 0 0 1 0]	[0 0 0 0 1]

**Table 5:** Training sample data for load identification in BP neural network.

**Type**	**T1**	**T2**	**T3**	**T4**	**T5**
impact (0.5 s)	0.0965	0.0927	0.0664	0.0988	0.0927
steady	0.1285	0.1354	0.1038	0.0631	0.1170
linear	0.0755	0.0853	0.0919	0.0779	0.1066
sinusoidal	0.1624	0.1034	0.0766	0.0889	0.0843
transient (3 s)	0.1874	0.1415	0.0875	0.0771	0.0721
**Type**	**T6**	**T7**	**T8**	**T9**	**T10**
impact (0.5 s)	0.1104	0.0826	0.1082	0.1081	0.1436
steady	0.1169	0.1069	0.0738	0.0746	0.0800
linear	0.0837	0.0755	0.096	0.1271	0.1805
sinusoidal	0.0996	0.0900	0.0965	0.0881	0.1102
transient (3 s)	0.0865	0.0922	0.0771	0.0937	0.0849

**Table 6:** Texting sample data for load identification in BP neural network (sinusoidal load).

**No.**	**T1**	**T2**	**T3**	**T4**	**T5**
1	0.1336	0.1044	0.0903	0.1100	0.0965
2	0.7920	0.0785	0.0164	0.0032	0.0050
3	0.0924	0.0970	0.1131	0.0863	0.1096
**No.**	**T6**	**T7**	**T8**	**T9**	**T10**
1	0.1150	0.0954	0.0878	0.0788	0.0882
2	0.0048	0.0111	0.0190	0.0382	0.0318
3	0.0999	0.0917	0.1066	0.0897	0.1137
